# Supplementary material for: The use of Kudoh method for culture of Mycobacterium tuberculosis and Mycobacterium africanum in The Gambia
Source: PLoS One. 2024 Mar 27;19(3):e0300042. doi: 10.1371/journal.pone.0300042 (PMC10971581; doi:10.1371/journal.pone.0300042)
Supplement: Attachment — Submitted filename: Jobarteh et al 2016.pdf [file pone.0300042.s002.pdf]

Available at www.sciencedirect.com
ScienceDirect
journal homepage: www.elsevier.com/locate/IJMYCO

Evaluation of the Kudoh method for mycobacterial culture: Gambia experience

Tijan Jobarteh [a,*], *Jacob Otu* [a], *Ensa Gitteh* [a], *Francis Mendy* [a], *Tutty I. Faal-Jawara* [a], *Boatema Ofori-Anyinam* [a], *Abigail Ayorinde* [a], *Ousman Secka* [a], *Martin Antonio* [a], *Florian Gehre* [a,b]

[a] *Medical Research Council Unit, Gambia*
[b] *Institute of Tropical Medicine, Antwerp, Belgium*

ARTICLE INFO

Article history:
Received 18 September 2016
Accepted 20 September 2016
Available online 11 November 2016

Keywords:
Kudoh method
Lowenstein Jensen medium
Modified Ogawa medium
Mycobacteria
N-Acetyl-L-cysteine-NaOH method
Tuberculosis

ABSTRACT

Objective/background: To evaluate the Kudoh swab method for improving laboratory diagnosis of tuberculosis (TB) in Gambia.
Methods: A total of 75 sputa (50 smear positive and 25 smear negative) were examined. Sputum samples were collected from leftover routine samples from the Medical Research Council Unit, Gambia TB Diagnostic Laboratory. The samples were processed using the standard N-acetyl-L-cysteine-NaOH (NALC-NaOH) methods currently used and Kudoh swab method. These were cultured on standard Lowenstein Jensen (LJ) and Modified Ogawa media, respectively, and incubated aerobically at 36 ± 1 °C for mycobacterial growth. To determine if the decontamination and culture methods compared could equally detect the *Mycobacterium tuberculosis* complex (MTBC) highly commonly isolated in Gambia, spoligotyping was done.
Results: In total, 72% (54/75) of MTBC were recovered by both LJ and Modified Ogawa methods. The LJ method recovered 52% (39/75) and Modified Ogawa recovered 56% (42/75) of the MTBC, respectively. Spoligotyping showed Euro-American 35% (19/54), Indo-Oceanic 35% (19/54), *Mycobacterium africanum* (West African type 2) 26% (14/54), Beijing 2% (1/54), and *M. africanum* (West African type 1) 2% (1/54).
Conclusion: The Kudoh method is simpler and cheaper than the NALC-NaOH method. There was no significant difference in recovery between the methods. The Kudoh method is ideal in overburdened TB laboratories with poor resources in developing countries. The predominant lineages were Euro-American and Indo-Oceanic, followed by *M. africanum* (West African type 2).

Conflict of interest

The authors have no conflict of interest to declare.

* Corresponding author at: Medical Research Council Unit, The Gambia Atlantic Boulevard, Fajara, P. O. Box 273, Banjul, Gambia.
E-mail address: tijanjobarteh5@hotmail.com (T. Jobarteh).
Peer review under responsibility of Asian African Society for Mycobacteriology.
http://dx.doi.org/10.1016/j.ijmyco.2016.09.049
